# Optical Coherence Tomography Angiography in Type 1 Diabetes Mellitus. Report 1: Diabetic Retinopathy

**DOI:** 10.1167/tvst.9.10.34

**Published:** 2020-09-30

**Authors:** Marina Barraso, Aníbal Alé-Chilet, Teresa Hernández, Cristian Oliva, Irene Vinagre, Emilio Ortega, Marc Figueras-Roca, Anna Sala-Puigdollers, Cristina Esquinas, Enric Esmatjes, Alfredo Adán, Javier Zarranz-Ventura

**Affiliations:** 1 Institut Clínic d'Oftalmologia (ICOF), Hospital Clínic, Barcelona, Spain; 2 Diabetes Unit, Institut Clínic de Malalties Digestives i Metabòliques (ICMDM), Hospital Clínic, Barcelona, Spain; 3 Respiratory Department, Hospital Universitari Vall d'Hebron, Barcelona, Spain; 4 Centro de Investigación Biomédica en Red de la Fisiopatología de la Obesidad y Nutrición (CIBEROBN), Spain; 5 August Pi i Sunyer Biomedical Research Institute (IDIBAPS), Barcelona, Spain

**Keywords:** diabetic retinopathy, optical coherence tomography angiography, retinal blood flow, type 1 diabetes mellitus, retinal imaging

## Abstract

**Purpose:**

The purpose of this study was to evaluate specifically in type 1 diabetes mellitus (DM) individuals the relationship between perifoveal superficial capillary plexus (SCP) parameters assessed by optical coherence tomography angiography (OCTA) and diabetic retinopathy (DR) grade.

**Methods:**

Cross-sectional analysis of a large scale prospective OCTA trial cohort (ClinicalTrials.gov NCT03422965). A total of 1186 eyes (593 individuals), 956 type 1 DM eyes (478 patients), and 230 control eyes (115 healthy volunteers) were included in this study. DR stage was graded according to the International Classification. OCTA imaging was performed with a commercially available device (Cirrus HD-OCT). Vessel density (VD), perfusion density (PD), and foveal avascular zone (FAZ) area, perimeter and circularity measurements were quantified in the SCP and receiver operating characteristic (ROC) curves were constructed for each OCTA parameter.

**Results:**

VD and PD (in both 3 × 3 and 6 × 6 mm captures) were inversely associated with DR stage (*P* < 0.001 in all cases) in a multiple regression analysis after controlling by age, gender, signal strength index, axial length, and DM duration. Greater FAZ area and perimeter and conversely lower circularity measurements were observed as DR severity increased in both scanning protocols (*P* < 0.05 in all cases).

**Conclusions:**

In type 1 DM individuals, OCTA provides an objective, continuous, and reliable method for accurate quantification of VD, PD, and FAZ parameters in the SCP, which ultimately correlate with DR stages.

**Translational Relevance:**

Objective OCTA measurements of the retinal microvasculature could substitute the clinical DR classification in patients with type 1 DM, identify patients at risk of DR progression, and inform treatment decisions to modify the evolution of the disease.

## Introduction and Purpose

Diabetic retinopathy (DR) is a clinically diverse entity originating from the alteration of small retinal vessels. A selective loss of pericytes and endothelial cells, as well as the thickening of the basal membrane in retinal capillaries, occurs as a result of a long period of exposure to elevated glucose levels in blood, damaging the retinal vessels and affecting the retinal flow in a time dependent manner.^1^ Until recently, the only technique that permitted the evaluation of vascular flow in the retina of patients with diabetes mellitus (DM) was fluorescein angiography (FA), a test that requires an intravenous injection of dye.^2^ Because this technique cannot visualize or quantify separately the main two major networks of capillaries, the superficial capillary plexus (SCP) and deep capillary plexus (DCP), its role in the understanding of selective vessel plexus implication in DR appears limited. For these two reasons, in the research setting, FA has been relegated by optical coherence tomography angiography (OCTA), a novel noninvasive retinal imaging procedure.^3^

OCTA represents an interesting opportunity to evaluate the perifoveal vascular network at different moments of the disease, with the potential of being an excellent tool to identify early changes (capillary dilatation, foveal avascular zone [FAZ] enlargement, impaired capillary perfusion in paramacular areas, or presence of microaneurysms) in a noninvasive way.^3^^,^^4^ Many studies have been carried out in the last years to evaluate the perifoveal vascular flow and FAZ features in patients with diabetes using OCTA. However, the vast majority represent a relatively short series of patients, they rarely have a control group, and, more importantly, almost all of them have been performed in patients with type 2 DM. Type 2 DM has a completely different pathophysiology than type 1 DM, and it commonly affects older patients with other comorbidities, such as hypertension or dyslipidemia, that may affect the quantification of OCTA images.

With this aim, we designed a prospective research project directed to study the retinal vascular network through OCTA in a large cohort of patients with type 1 DM^5^ (registered in ClinicalTrials.gov, NCT03422965). In agreement with the Diabetes Unit of the Endocrinology Department, the routine yearly fundus retinography of patients with type 1 DM was replaced by a comprehensive ocular examination, which included an extensive battery of OCT and OCTA image collection. Associations between ocular data derived from this examination and demographic and systemic data from current and last 5 years blood test data were evaluated, with the objective to identify possible biomarkers of the disease.

The purpose of this specific report is to determine objectively in a cross-sectional analysis the characteristics of the perifoveal capillary network in patients with type 1 DM and to study their relationship with the DR grade compared to those observed in a control group of healthy volunteers. We aim to evaluate the potential of OCTA as a new tool to detect objective parameters of DM status in the perifoveal vascular network in a large series of patients with type 1 DM, which may have direct implications in the systemic management of these patients.

## Methods

### Study Design and Study Protocol

A cross sectional, exploratory study was conducted in a large cohort of patients with type 1 DM recruited from the Diabetes Unit of Hospital Clínic in a 24-month period (May 2017–May 2019), with prospective collection of OCTA images and relevant ocular and systemic clinical data. The study protocol has been described elsewhere.^5^ This project was approved by the Institutional Review Board of Hospital Clinic of Barcelona (study protocol version 0.2, 23/11/2016) and it is registered in the Clinical Trials website (ClinicalTrials.gov NCT03422965). Written informed consent was obtained for all participants.

### Inclusion and Exclusion Criteria

**Figure 1.**
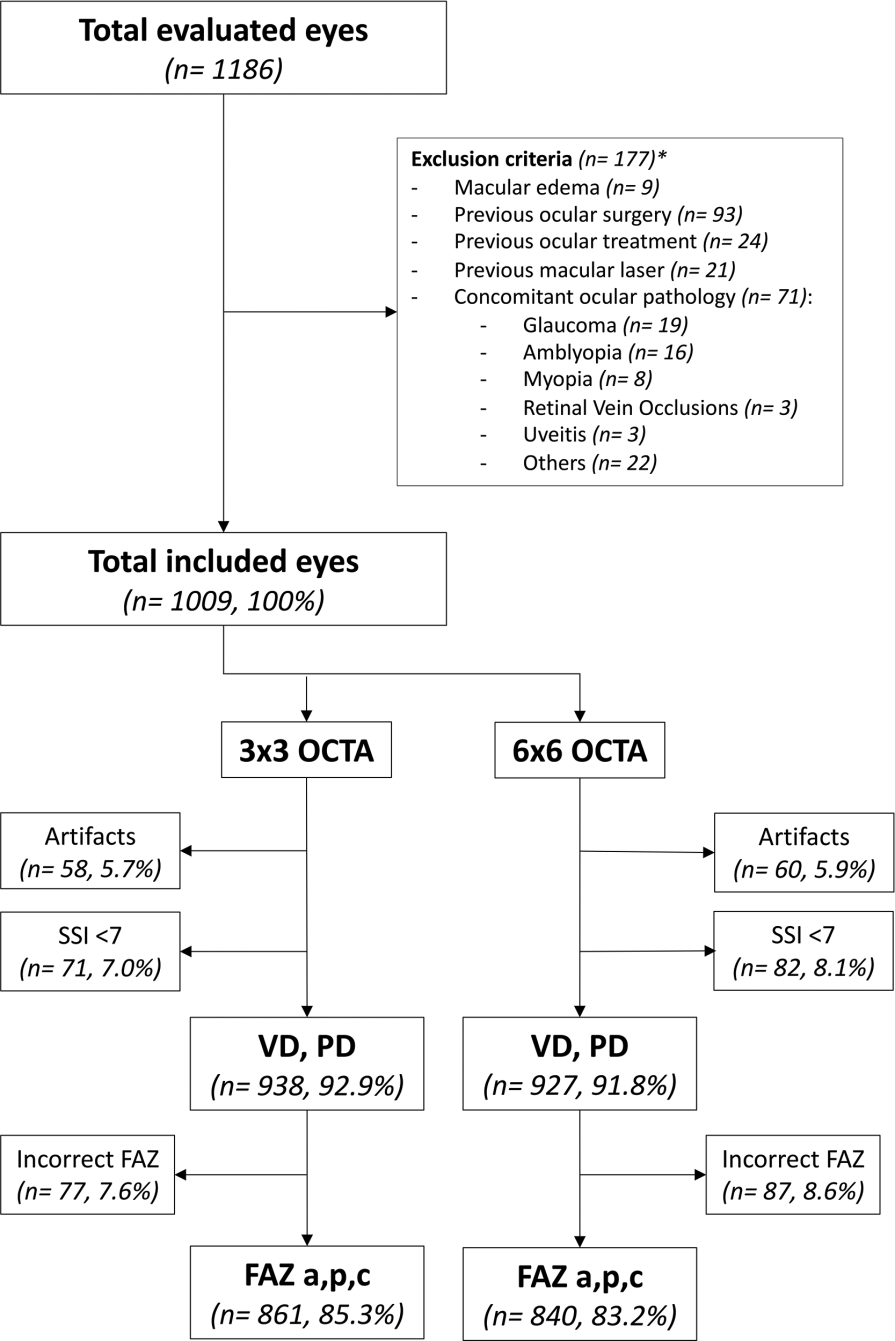
Consolidated standard of reporting trials (CONSORT)-style flow chart describing included and excluded eyes in the study and each individual optical coherence tomography angiography (OCTA) analysis. (*1 eye = ≥ 1 criteria for exclusion; VD = vessel density; PD = perfusion density; FAZ = foveal avascular zone; a = area; p = perimeter; c = circularity; SSI = signal strength index).

Patients with type 1 DM undergoing yearly follow-up visits, as per routine clinical care at the Diabetes Unit of our center, were invited to participate in the study. Those willing to participate were referred for a comprehensive ocular examination. Controls were collected from healthy volunteers recruited in the general population after social media campaigns supported by the Communications department of the hospital. Exclusion criteria include concomitant ocular pathologies, macular edema, presence of macular cysts, previous ocular surgery, previous macular laser, previous ocular treatment, including intravitreal therapies, media opacities, and inability to perform complete ocular examinations, including retinal imaging (OCT, OCTA, fundus retinographies, biometry, etc.), as well as inability to give written informed consent to participate in the study. A consolidated standard of reporting trials (CONSORT)-style flow chart describing included and excluded eyes in the study and each individual OCTA analysis is presented in Figure 1.

### Interventions: Ocular and Systemic Data

All participants underwent a complete ocular examination. Clinical data collected included: best-corrected visual acuity (BCVA), slit-lamp biomicroscopy, intraocular pressure measurement, retinal fundus examination, and biometry (IOL Master, Carl Zeiss Meditec, Dublin, CA). Diabetic retinopathy stage was graded in all study eyes using the International Clinical Diabetic Retinopathy Disease Severity Scale.^6^ A comprehensive battery of OCT and OCTA images was performed as described below.

Systemic status of the diabetic disease was evaluated in routine clinical care examinations in the Diabetes Unit of the Endocrinology service, and clinical data collected included: age, gender, smoking habit, duration of the disease, type of treatment (insulin doses, lipid-lowering, antihypertensive, and antiplatelet drugs), concomitant pathology (hypertension, dyslipidemia, and macrovascular complications), body mass index (BMI), hemogram, HbA1c, lipid profile (total cholesterol, triglycerides, high-density lipoprotein [HDL] cholesterol, low-density lipoprotein [LDL] cholesterol), and kidney function tests (creatinine and urinary albumin excretion).

### OCTA and OCT Imaging Protocols

**Figure 2.**
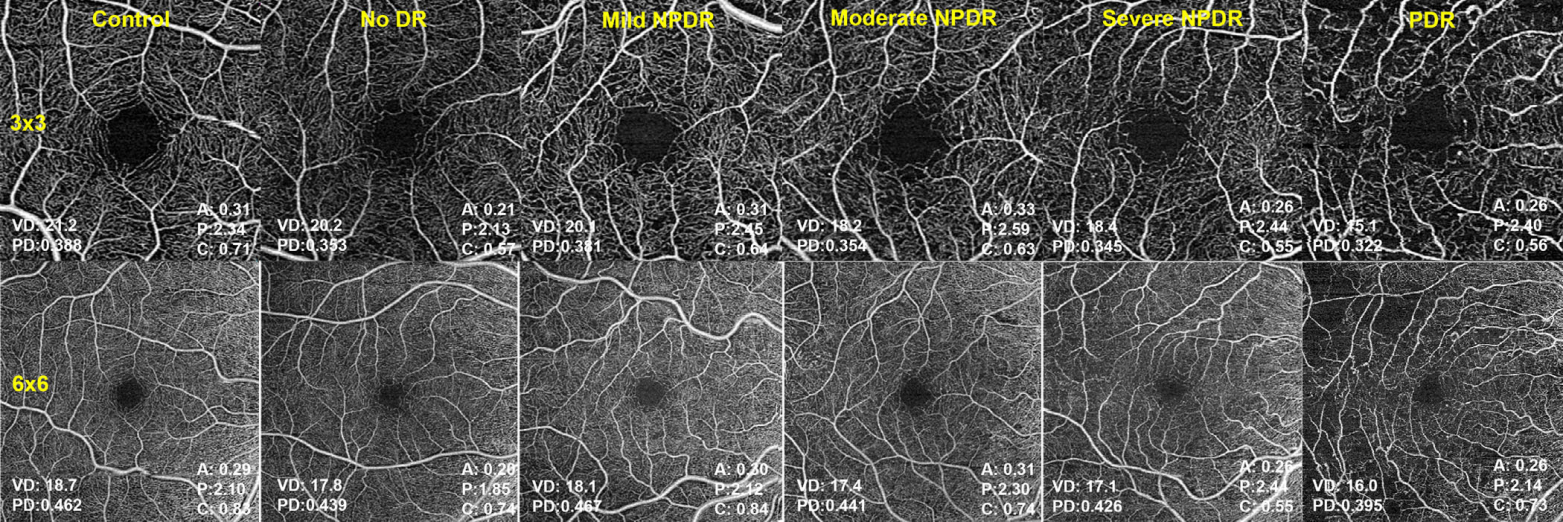
Optical coherence tomography angiography images of the superficial capillary plexus in study eyes. Examples of 3 × 3 mm (top row) and 6 × 6 mm (bottom row) OCTA captures of the control group (left) and different diabetic retinopathy (DR) stages showing progressively lower vessel density (VD) and perfusion density (PD), greater foveal avascular zone (FAZ) area. and perimeter and lower FAZ circularity in greater DR stages.

All OCTA and OCT images were obtained using the same OCT device (Cirrus HD-OCT model 5000; Carl Zeiss Meditec, Dublin, CA). OCTA scanning protocols included 3 × 3 mm and 6 × 6 mm cube scans centered in the fovea by gaze fixation (Fig. 2). Structural OCT protocol included a macular cube scan (512 × 128). OCTA and OCT images with presence of artifacts, segmentation errors, or a signal strength index (SSI) < 7 were excluded from analysis. OCTA quantifications were performed by the device built-in commercial software (AngioPlex Metrix, Carl Zeiss Meditec, Dublin, CA) only in the SCP of the study eyes, defined as the layer between the internal limiting membrane (ILM) and the inner plexiform layer (IPL) boundaries. AngioPlex Metrix measurements included vessel and perfusion density and FAZ parameters (area, perimeter, and circularity) displayed for 3 × 3 mm and 6 × 6 mm scans. Vessel density is the total length of perfused vasculature per unit area in the region of measurement; perfusion density is the total area of perfused vasculature per unit area in the region of measurement. No manual adjustments of the segmentation slab boundaries were performed during the conduction of this study. A detailed description of OCTA images included and excluded from analysis is presented in Figure 1.

### Statistical Analysis

In order to describe the qualitative variables, absolute frequencies and percentages were used. The description of quantitative variables was performed using the mean, standard deviation (SD), median, and quartiles. The Kolmogorov-Smirnov test was used to assess the normality of distributions. Clinical variables, OCTA, and structural parameters according to the DR grade were compared using a Generalized Estimating Equation (GEE) in order to control the potential bias of bilaterality and adjusted by age, gender, SSI, axial length, and DM disease duration from diagnosis. ANOVA or Kruskal-Wallis test (if normality was not assumed) with Bonferroni correction was used in multiple comparisons between groups in the case of quantitative variables. The analysis at the patient level was analyzed using linear regression for continuous variables or Mantel-Hansel test for categorical variables. Receiver operating curves (ROC) were constructed to evaluate the area under the curve (AUC) of each OCTA parameter by subgroups (controls, no diabetic retinopathy, diabetic retinopathy). For all the tests, *P* values < 0.05 were considered statistically significant. The statistical package R Studio (version 2.5) was used for the statistical analyses.

## Results

A total number of 1186 of eyes of 593 individuals were originally included in the study. Four hundred seventy-eight patients with type 1 DM (956 eyes) and 115 healthy controls (230 eyes) underwent a complete ocular examination during the predetermined timeframe. Exclusion criteria were applied and 181 eyes were excluded due to previous ocular surgery (93 eyes), previous ocular treatment (24 eyes), previous macular laser (21 eyes), macular edema (9 eyes), or concomitant ocular pathology (71 eyes), such as glaucoma (19 eyes), amblyopia (16 eyes), myopia magna, or axial length > 27 mm (8 eyes), and other causes (28 eyes), such as retinal vein occlusions or uveitis. OCTA images from 1009 eyes were analyzed. A detailed description of OCTA images excluded due to artifacts, poor OCTA image quality (defined as SSI < 7), or incorrect FAZ delineation by the built-in software for each OCTA parameter analysis is presented in Figure 1.

Mean age for patients with diabeteswas 40 ± 12.4 (standard deviation [SD]) years and the mean disease duration was 20 ± 10.8 years. Mean age for healthy controls was 43 ± 14.1 years. All demographic characteristics are shown in Supplementary Table S1. The distribution of DR grades was no DR in 53.4% (*n* = 539) of the examined eyes, mild non-proliferative diabetic retinopathy (NPDR) in 21.2% (*n* = 214), moderate NPDR in 3.3% (*n* = 34), severe NPDR in 0.4% (*n* = 5), and finally proliferative DR (previously treated with panretinal photocoagulation in all cases) in 1.3% (*n* = 14) of the study eyes. All baseline characteristics of study eyes according to DR stage distribution are summarized in Supplementary Table S2. All OCTA measurements were controlled and adjusted by diabetic retinopathy grade baseline characteristics in a multivariate analysis.

### Vessel Density, Perfusion Density, and Diabetic Retinopathy Grade

**Figure 3.**
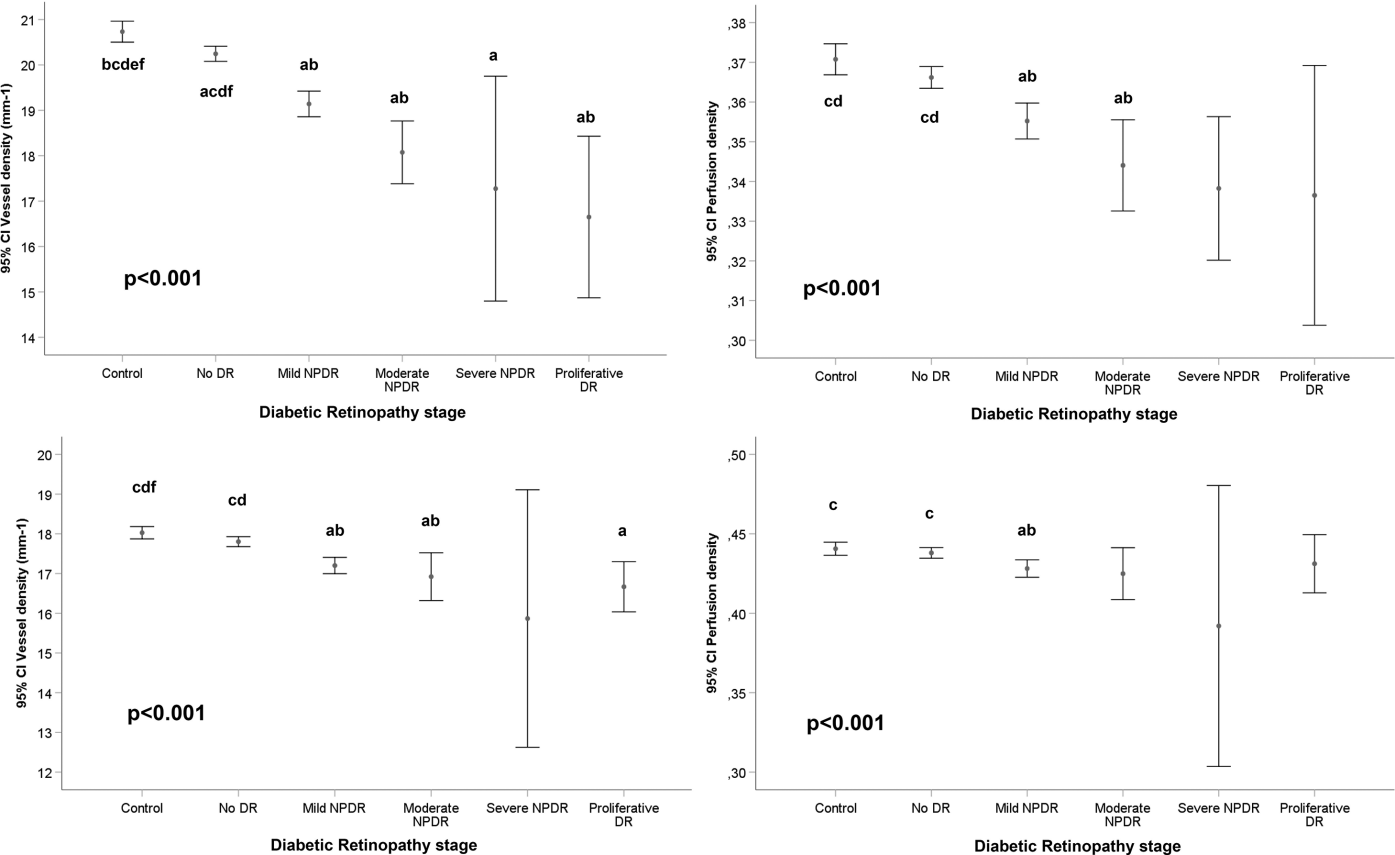
Vessel density (VD) and perfusion density (PD) measurements and diabetic retinopathy (DR) grade, after adjusting for age, gender, signal strength index (SSI), axial length and duration of diabetes mellitus disease. *Top*: VD (*left*) and PD (*right*) measurements in the complete scanned area using the 3 × 3 mm scanning protocol. *Bottom*: VD (*left*) and PD (*right*) measurements in the complete scanned area using the 6 × 6 mm scanning protocol. Generalized Estimating Equation (GEE) adjusted for age, gender, signal strength index (SSI), axial length, and duration of diabetes mellitus disease for global *P* value; pairwise comparisons by Kruskal-Wallis test with Bonferroni correction. *P* < 0.05: (a**)** vs. Control; (**b**) vs. No DR; (**c**) vs. Mild DR; (**d**) vs. Moderate DR; (**e**) vs. Severe DR; (**f**) vs Proliferative DR (Total number of eyes [3 × 3/6 × 6]: control 189/183, no DR 504/503, mild NPDR 202/198, moderate NPDR 29/30, severe NPDR 4/3, PDR 10/10).

**Figure 4.**
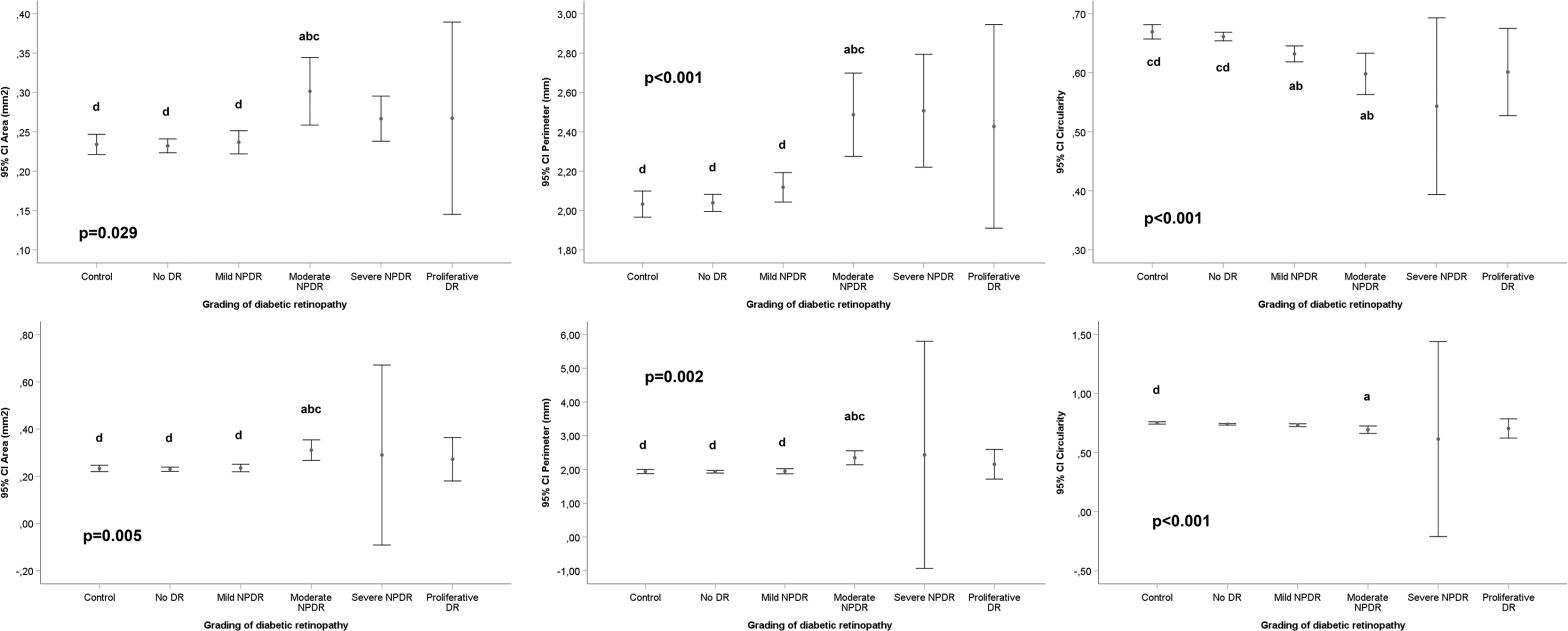
Foveal Avascular Zone (FAZ) measurements and diabetic retinopathy (DR) grade after adjusting for gender, age, SSI, axial length, and duration of diabetes mellitus disease. *Top*: FAZ area (*left*), perimeter (*middle*), and circularity (*right*) measurements in the complete scanned area using the 3 × 3 mm scanning protocol. *Bottom*: FAZ area (*left*), perimeter (*middle*), and circularity (*right*) measurements in the complete scanned area using the 6 × 6 mm scanning protocol. Generalized Estimating Equation (GEE) adjusted for age, gender, signal strength index (SSI), axial length, and duration of diabetes mellitus disease for global *P* value; pairwise comparisons by Kruskal-Wallis test with Bonferroni correction. *P* < 0.05: (**a**) vs. Control; (**b**) vs. No DR; (**c**) vs Mild DR; (**d**) vs Moderate DR; (**e**) vs Severe DR; (**f**) vs Proliferative DR. (Total number of eyes [3 × 3/6 × 6]: control 174/169, no DR 470/461, mild NPDR 180/178, moderate NPDR 26/26, severe NPDR 3/2, PDR 8/8).

Figure 2 depicts examples of 3 × 3 mm and 6 × 6 mm OCTA images of control eyes and different DR stages. The mean values of VD, perfusion density (PD), FAZ area, perimeter, and circularity in 3 × 3 and 6 × 6 mm captures in both diabetic and control patients are shown in Figure 3 and Figure 4 and in Table 1.

**Table 1.** OCTA-Derived Measurements of Study Eyes According to Diabetic Retinopathy Stage, Adjusted for Age, Gender, SSI, Axial Length, and Duration of Diabetes Mellitus Disease

		Total	Control	No DR	Mild NPDR	Moderate NPDR	Severe NPDR	Proliferative	*P* Value
Variable	Statistic	(*n* = 938)	(*n* = 189)	(*n* = 504)	(*n* = 202)	(*n* = 29)	(*n* = 4)	DR (*n* = 10)	for trend^a^
Vessel density 3 × 3	*n*	938	189	504	202	29	4	10	<0.001
(mm^−1^)	Mean (SD)	20 (1.9)	20.7 (1.6)	20.2 (1.7)	19.1 (1.9)	18.1 (1.6)	17.3 (1.6)	16.7 (1.7)	
	Median (IQR)	20.3 (19; 21.4)	21 (20.1; 22)	20.5 (19.3; 21.5)	19.4 (18.2; 20.3)	18.3 (17.5; 19.2)	17.8 (16.2; 18.4)	16.5 (16; 18.5)	
Perfusion density 3 × 3	*n*	938	189	504	202	29	4	10	<0.001
(0-1)	Mean (SD)	0.36 (0.03)	0.37 (0.03)	0.37 (0.03)	0.36 (0.03)	0.34 (0.03)	0.34 (0.01)	0.34 (0.03)	
	Median (IQR)	0.37 (0.35; 0.39)	0.38 (0.36; 0.39)	0.37 (0.35; 0.39)	0.36 (0.34; 0.38)	0.35 (0.34; 0.36)	0.34 (0.33; 0.35)	0.33 (0.32; 0.37)	
FAZ area 3 × 3	*n*	861	174	470	180	26	3	8	0.029
(mm^2^)	Mean (SD)	0.24 (0.1)	0.23 (0.09)	0.23 (0.1)	0.24 (0.1)	0.3 (0.11)	0.27 (0.01)	0.27 (0.15)	
	Median (IQR)	0.23 (0.17; 0.3)	0.24 (0.17; 0.29)	0.22 (0.17; 0.29)	0.23 (0.17; 0.31)	0.32 (0.25; 0.39)	0.26 (0.26; 0.28)	0.29 (0.14; 0.4)	
FAZ perimeter 3 × 3	*n*	861	174	470	180	26	3	8	<0.001
(mm)	Mean (SD)	2.07 (0.49)	2.03 (0.45)	2.04 (0.48)	2.12 (0.52)	2.49 (0.54)	2.51 (0.12)	2.43 (0.62)	
	Median (IQR)	2.09 (1.78; 2.4)	2.09 (1.78; 2.4)	2.05 (1.77; 2.4)	2.14 (1.77; 2.5)	2.59 (2.2; 2.8)	2.44 (2.44; 2.6)	2.69 (2.23; 2.8)	
FAZ circularity 3 × 3	*n*	861	174	470	180	26	3	8	<0.001
(0-1)	Mean (SD)	0.65 (0.09)	0.67 (0.08)	0.66 (0.08)	0.63 (0.09)	0.6 (0.09)	0.54 (0.06)	0.6 (0.09)	
	Median (IQR)	0.66 (0.6; 0.71)	0.68 (0.62; 0.73)	0.67 (0.61; 0.72)	0.65 (0.58; 0.7)	0.58 (0.54; 0.67)	0.55 (0.48; 0.6)	0.61 (0.53; 0.69)	
Vessel density 6 × 6	*n*	927	183	503	198	30	3	10	<0.001
(mm^−1^)	Mean (SD)	17.7 (1.3)	18 (1.1)	17.8 (1.3)	17.2 (1.3)	16.9 (1.4)	15.9 (1.3)	16.7 (0.6)	
	Median (IQR)	18 (17; 18.6)	18.3 (17.6; 18.7)	18.2 (17.3; 18.7)	17.5 (16.6; 18.2)	17.3 (16.5; 17.9)	16 (14.5; 17.1)	16.8 (16.3; 17.1)	
Perfusion density 6 × 6	*n*	927	183	503	198	30	3	10	<0.001
(0-1)	Mean (SD)	0.44 (0.03)	0.44 (0.03)	0.44 (0.03)	0.43 (0.04)	0.42 (0.04)	0.39 (0.04)	0.43 (0.02)	
	Median (IQR)	0.45 (0.42; 0.46)	0.45 (0.43; 0.46)	0.45 (0.43; 0.46)	0.44 (0.41; 0.45)	0.44 (0.41; 0.45)	0.4 (0.36; 0.43)	0.43 (0.42; 0.44)	
FAZ area 6 × 6	*n*	840	169	461	178	26	2	8	0.005
(mm^2^)	Mean (SD)	0.24 (0.1)	0.23 (0.08)	0.23 (0.1)	0.24 (0.11)	0.31 (0.12)	0.29 (0.04)	0.27 (0.13)	
	Median (IQR)	0.23 (0.17; 0.3)	0.24 (0.19; 0.29)	0.22 (0.16; 0.29)	0.21 (0.16; 0.3)	0.33 (0.24; 0.39)	0.29 (0.26; 0.32)	0.31 (0.15; 0.39)	
FAZ perimeter 6 × 6	*n*	840	169	461	178	26	2	8	0.002
(mm)	Mean (SD)	1.96 (0.46)	1.94 (0.4)	1.93 (0.44)	1.95 (0.51)	2.35 (0.57)	2.44 (0.37)	2.16 (0.61)	
	Median (IQR)	1.97 (1.67; 2.3)	2 (1.76; 2.2)	1.93 (1.66; 2.2)	1.95 (1.64; 2.3)	2.4 (2.04; 2.8)	2.44 (2.17; 2.7)	2.34 (1.72; 2.5)	
FAZ circularity 6 × 6	*n*	840	169	461	178	26	2	8	<0.001
(0-1)	Mean (SD)	0.74 (0.08)	0.75 (0.06)	0.74 (0.07)	0.73 (0.08)	0.69 (0.09)	0.62 (0.09)	0.71 (0.11)	
	Median (IQR)	0.75 (0.69; 0.79)	0.76 (0.72; 0.8)	0.76 (0.7; 0.79)	0.75 (0.69; 0.8)	0.72 (0.63; 0.76)	0.62 (0.55; 0.68)	0.7 (0.66; 0.8)	
Mean RNFL	*n*	943	188	511	200	30	4	10	0.180
(microns)	Mean (SD)	96.3 (10.2)	96.5 (9.2)	96.7 (9.9)	95.4 (11.3)	99.3 (10.9)	84.8 (13.1)	89.5 (13.3)	
	Median (IQR)	96 (90; 103)	95 (90; 104)	97 (91; 103)	96 (89; 104)	100 (93; 105)	84 (73.5; 96)	86 (81; 87)	
Central macular thickness	*n*	959	188	516	207	34	4	10	0.339
(microns)	Mean (SD)	262.6 (21.5)	262.8 (22.2)	261.5 (19.9)	264.2 (21.8)	269.5 (30.5)	281 (21.5)	244.8 (39.4)	
	Median (IQR)	262 (248; 277)	261 (247; 280)	262 (249; 275)	265 (250; 278)	268 (246; 286)	280.5 (262.5; 299)	230 (228; 266)	
Central macular volume	*n*	959	188	516	207	34	4	10	0.716
(microns^3^)	Mean (SD)	10.3 (0.6)	10.3 (0.5)	10.3 (0.4)	10.3 (0.9)	10.4 (0.6)	10.1 (0.9)	9.8 (0.8)	
	Median (IQR)	10.3 (10; 10.6)	10.3 (9.9; 10.6)	10.3 (10; 10.6)	10.2 (9.9; 10.5)	10.3 (10; 10.8)	10.3 (9.4; 10.8)	9.9 (9.7; 10.1)	
Mean central macular thickness	*n*	959	188	516	207	34	4	10	0.387
(microns)	Mean (SD)	284.6 (17.6)	285.3 (14.9)	284.6 (20.2)	283.9 (12.9)	288 (17.3)	278.8 (25.2)	272.6 (22.5)	
	Median (IQR)	285 (277; 294)	285 (274; 295)	286 (279; 293)	283 (275; 292)	285.5 (276; 298)	285 (259; 298.5)	276 (269; 280)	

OCTA, optical coherence tomography angiography; DR, diabetic retinopathy; NPDR, nonproliferative diabetic retinopathy; FAZ, foveal avascular zone; SD, standard deviation; IQR, interquartile range.

a Generalized Estimating Equation (GEE) adjusted for age, gender, signal strength index (SSI), axial length, and duration of diabetes mellitus disease.

In 3 × 3 mm captures, a significant association was observed between VD and DR stage, showing lower VD with greater levels of DR stage and statistically significant differences between the different disease severity groups and controls (*P* < 0.001), after adjusting data by age, gender, SSI, axial length, and duration of DM disease. Differences between each different DR groups are detailed in Figure 3. PD also showed significant differences (*P* < 0.001 in all cases) between the DR stage groups with lower PD in greater levels of DR severity. Similar findings were observed with the 6 × 6 mm captures, as shown in Figure 3 and Table 1.

### Foveal Avascular Zone Measurements and Diabetic Retinopathy Grade

FAZ parameters showed different results in 3 × 3 and 6 × 6 mm scans, as shown in Figure 4. In 3 × 3 mm scans, significant differences were observed in the FAZ area (*P* = 0.029), FAZ perimeter (*P* < 0.001), and FAZ circularity (*P* < 0.001) between controls and the different DR severity groups. Statistically significant differences (*P* < 0.05) were found in 2 × 2 comparisons between controls and some of the DR groups, as well as between some of the DR groups, as detailed in Figure 4. Similarly, in 6 × 6 mm scans significant differences between controls and the DR groups were observed for FAZ area (*P* = 0.005), FAZ perimeter (*P* = 0.002), and FAZ circularity (*P* < 0.001). Statistically significant differences were found in 2 × 2 comparisons between controls and some of the DR groups (*P* < 0.05), as well as between some of the different DR groups. All these results are summarized in Figure 4 and Table 1.

### Influence of Diabetes Mellitus Duration on OCTA Metrics

Subgroup analysis was performed by duration of DM and groups were defined as A (< 5 years), B (5–15 years), and C (> 15 years). Significant differences were observed in the 3 × 3 mm scans for VD (20.8 ± 1.5 vs. 20.7 ± 1.5 vs. 19.3 ± 1.9, *P* < 0.001) and PD (0.37 ± 0.02 vs. 0.37 ± 0.03 vs. 0.36 ± 0.03, *P* < 0.001) for these 3 subgroups in a lineal regression model adjusted for age, gender, SSI, axial length, and DR grade. With regard to the FAZ parameters, no differences were observed for FAZ area (0.24 ± 0.08 vs. 0.23 ± 0.1 vs. 0.23 ± 0.1, *P* = 0.52) or FAZ perimeter (2.1 ± 0.38 vs. 2.01 ± 0.47 vs. 2.11 ± 0.53, *P* = 0.146), but significant differences were observed in FAZ circularity (0.67 ± 0.08 vs. 0.67 ± 0.07 vs. 0.64 ± 0.09, *P* = 0.01). Similar results were observed in the 6 × 6 mm scans, and differences were observed for VD (18.1 ± 1.2 vs. 18.0 ± 1.2 vs. 17.3 ± 1.4, *P* < 0.001), PD (0.44 ± 0.03 vs. 0.44 ± 0.03 vs. 0.43 ± 0.04, *P* < 0.001) for these 3 subgroups, in a lineal regression model adjusted for age, gender, SSI, axial length, and DR grade. No differences were observed for FAZ area (0.24 ± 0.09 vs. 0.23 ± 0.09 vs. 0.24 ± 0.11, *P* = 0.541) and FAZ perimeter (1.96 ± 0.4 vs. 1.94 ± 0.43 vs. 1.97 ± 0.51, *P* = 0.537) but FAZ circularity showed significant differences between groups (0.76 ± 0.07 vs. 0.74 ± 0.08 vs. 0.73 ± 0.08, *P* = 0.016). All these results are presented in Table 2.

**Table 2.** Influence of Diabetes Mellitus Duration on Optical Coherence Tomography Angiography (OCTA) Metrics

			Diabetes Mellitus Duration			
3 × 3mm OCTA	Statistic	Total (*N* = 605)	< 5 y (*N* = 49)	5-15 y (*N* = 165)	≥ 15 y (*N* = 391)	*P* Value^a^
Vessel density (mm-1)	Mean (SD)	19.8 (1.9)	20.8 (1.5)	20.7 (1.5)	19.3 (1.9)	< 0.001^b^
	Median (IQR)	20 (18.7; 21.2)	20.8 (19.9; 22)	21 (20; 21.8)	19.5 (18.2; 20.6)	
Perfusion density	Mean (SD)	0.36 (0.03)	0.37 (0.02)	0.37 (0.03)	0.36 (0.03)	< 0.001^b^
	Median (IQR)	0.37 (0.35; 0.38)	0.38 (0.36; 0.39)	0.38 (0.36; 0.39)	0.36 (0.34; 0.38)	
Area (mm2)	Mean (SD)	0.24 (0.1)	0.24 (0.08)	0.23 (0.1)	0.24 (0.1)	0.523
	Median (IQR)	0.23 (0.17; 0.3)	0.24 (0.17; 0.29)	0.22 (0.17; 0.28)	0.23 (0.17; 0.31)	
Perimeter (mm)	Mean (SD)	2.08 (0.5)	2.1 (0.38)	2.01 (0.47)	2.11 (0.53)	0.146
	Median (IQR)	2.09 (1.79; 2.4)	2.07 (1.86; 2.4)	2.01 (1.77; 2.4)	2.16 (1.79; 2.5)	
Circularity	Mean (SD)	0.65 (0.09)	0.67 (0.08)	0.67 (0.07)	0.64 (0.09)	0.001 ^b^
	Median (IQR)	0.66 (0.6; 0.71)	0.68 (0.62; 0.74)	0.67 (0.62; 0.72)	0.65 (0.58; 0.7)	
6 × 6mm OCTA	Statistic	Total (*N* = 598)	Diabetes Mellitus Duration			*P* Value^a^
			< 5 y (*N* = 49)	5-15 y (*N* = 161)	≥ 15 y (*N* = 388)	
Vessel density (mm-1)	Mean (SD)	17.6 (1.3)	18.1 (1.2)	18 (1.2)	17.3 (1.4)	< 0.001^b^
	Median (IQR)	17.9 (16.9; 18.6)	18.5 (17.7; 18.9)	18.3 (17.7; 18.8)	17.6 (16.6; 18.3)	
Perfusion density	Mean (SD)	0.43 (0.04)	0.44 (0.03)	0.44 (0.03)	0.43 (0.04)	< 0.001^b^
	Median (IQR)	0.45 (0.42; 0.46)	0.45 (0.44; 0.46)	0.45 (0.43; 0.46)	0.44 (0.41; 0.46)	
Area (mm2)	Mean (SD)	0.24 (0.1)	0.24 (0.09)	0.23 (0.09)	0.24 (0.11)	0.541
	Median (IQR)	0.23 (0.16; 0.3)	0.24 (0.18; 0.29)	0.22 (0.17; 0.29)	0.23 (0.16; 0.3)	
Perimeter (mm)	Mean (SD)	1.96 (0.48)	1.96 (0.4)	1.94 (0.43)	1.97 (0.51)	0.537
	Median (IQR)	1.97 (1.66; 2.3)	2 (1.71; 2.2)	1.89 (1.67; 2.2)	1.98 (1.64; 2.3)	
Circularity	Mean (SD)	0.73 (0.08)	0.76 (0.07)	0.74 (0.08)	0.73 (0.08)	0.016
	Median (IQR)	0.75 (0.69; 0.79)	0.77 (0.73; 0.82)	0.75 (0.69; 0.8)	0.74 (0.69; 0.79)	

SD, indicates standard deviation; IQR, interquartile range.

a Generalized Estimating Equation (GEE). Unadjusted *P* value.

b Generalized Estimating Equation (GEE). Adjusted *P* value < 0.05 (adjusted for age, gender, signal strength index (SSI), axial length, and diabetic retinopathy stage).

### Receiver Operating Curve Analysis of OCTA Parameters

**Figure 5.**
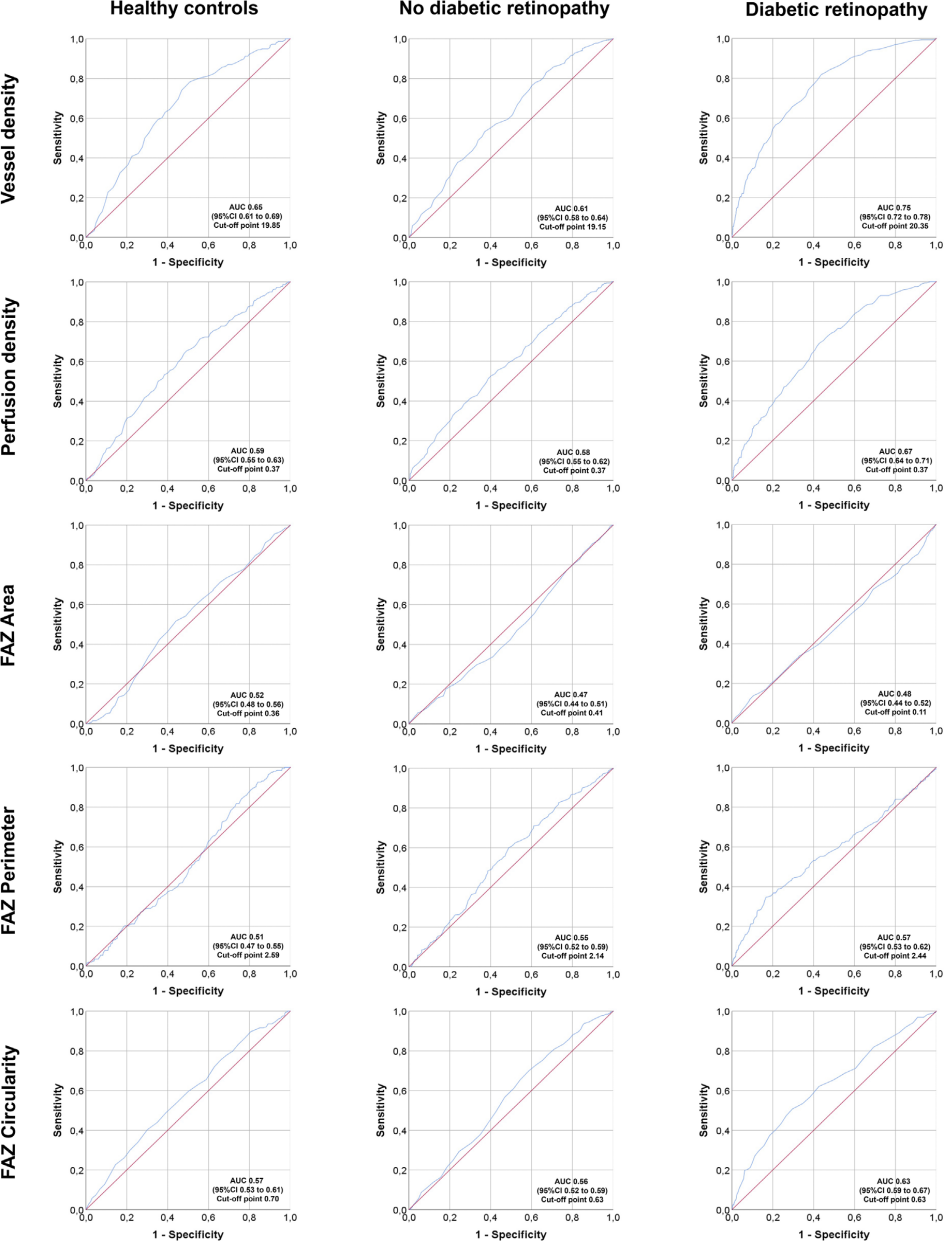
Receiver operating curves (ROC) of Optical Coherence Tomography Angiography (OCTA) parameters in 3 × 3 mm scans. Subgroup analysis by controls (*left column*), patients with type 1 diabetes mellitus with no diabetic retinopathy (*middle column*) and with diabetic retinopathy (mild, moderate, and severe nonproliferative and proliferative diabetic retinopathy)(*right column*). *Top row*: vessel density; *Top-middle row*: perfusion density; *Middle row*: Foveal Avascular Zone (FAZ) area; *Middle-bottom row*: FAZ perimeter; *Bottom row*: FAZ circularity. AUC, area under the curve; CI, confidence interval.

ROC were constructed to calculate the AUC for each one of the OCTA parameters in controls, patients with DM with no diabetic retinopathy, and patients with DM with diabetic retinopathy (non-proliferative and proliferative). The ROC curves for OCTA parameters in the 3 × 3 mm scans are presented in Figure 5. For VD, the AUC was 0.65, 0.61, and 0.75 in controls, no DR and DR. Proposed cut off points for VD in these subgroups are 19.85, 19.15, and 20.35, respectively. For PD, the AUC was 0.59, 0.58, and 0.67 in controls, no DR and DR. Proposed cut off points for PD in these subgroups are 0.37, 0.37, and 0.37, respectively. FAZ parameters were also evaluated with ROC curves. For FAZ area, the AUC was 0.52, 0.47, and 0.48 in controls, no DR and DR. Proposed cut off points for FAZ area in these subgroups are 0.36, 0.41, and 0.11, respectively. For FAZ perimeter, the AUC was 0.51, 0.55, and 0.57 in controls, no DR and DR. Proposed cut off points for FAZ perimeter in these subgroups are 2.59, 2.14, and 2.44, respectively. For FAZ circularity, the AUC was 0.57, 0.56, and 0.63 in controls, no DR and DR. Proposed cut off points for FAZ circularity in these subgroups are 0.70, 0.63, and 0.63, respectively.

## Discussion

This study highlights the role of OCTA as an objective tool to obtain reliable measurements of the foveal vessel status specifically in patients with type 1 DM, the rarest type of disease. These objective, quantitative, and continuous OCTA-derived measurements ultimately correlate with DR severity, showing gradually lower VD and PD, and greater FAZ changes in the SCP as DR progresses to greater levels of disease.

In the last few years, many OCTA studies have been directed to assess the status of the perifoveal capillary network in DM. However, the vast majority of studies have been performed in patients with type 2 DM,^7^^–^^15^ and many of them do not distinguish between patients with type 1 DM and patients with type 2 DM.^16^^–^^20^ The pathophysiology of both DM types is considerably different, and patients present well-differentiated clinical characteristics. Whereas type 1 DM is less frequent, commonly affects younger patients, and it is a lifelong standing disease, patients with type 2 DM are more prevalent, they tend to be older, and frequently present concomitant cardiovascular pathologies, such as blood hypertension, metabolic syndrome, or dyslipidemia. Given that age, duration of disease, and some of these other comorbidities have been associated to changes in OCTA metrics, these differences may affect either way the VD or PD measurements in patients with type 1 and type 2 diabetes. For these reasons, there is a paucity of data in the literature about OCTA measurements in type 1 DM eyes.

Very few papers have studied this topic specifically in patients with type 1 DM,^21^^–^^23^ and all of them included only a limited number of patients, as shown in Table 3. Carnevali et al. showed in a small series of 25 eyes that OCTA was able to detect early vascular alterations even without any biomicroscopic signs of DR.^24^ More recently, Sacconi et al. described a series of 34 eyes where the only OCTA parameter that was significantly different in patients with type 1 DM compared to controls was the PD of the DCP, without differences in the rest of the studied parameters and plexuses.^25^ The largest series in type 1 DM have been described in children, and they interestingly have shown controversial results.^26^^–^^30^ Golebiewska et al. reported that no differences were seen in the VD or FAZ area in any of the studied plexuses between patients with type 1 DM and controls (*n* = 188 vs. 60); however, Niestrata-Ortiz et al. described that FAZ area was different between diabetic and healthy children (*n* = 112 vs. 30).^27^^,^^28^ In this last paper, a greater FAZ area was also associated with a longer duration of the disease. Obviously, the mean age of the study cohorts in these pediatric series (15.3 and 13.8 years, respectively) needs to be considered when interpreting these results, in order to allow meaningful comparisons with adult populations.

**Table 3.** Selection of Relevant Papers Published to Date on OCTA and Diabetes Mellitus (*n* ≥ 25), Ordered by Diabetes Mellitus Type, Sample Size, and Publication Year

			Study Eyes							
		Type of		Type 1	Type 2		OCTA	Vascular	OCTA	
Author	Year	Diabetes	Total	DM	DM	Controls	Parameter	Plexus	Device	Software
Carnevali et al.^24^	2017	1	50	25	–	25	VD, FAZ	S, D, CC	Cirrus Angioplex	Custom - Image J
Sacconi et al.^25^	2019	1	61	34	–	27	PD, FAZ	S, D, CC	PLEX Elite 9000	Built in
Li et al.^26^	2019	1	91	47 (children)	–	44	VD, PD, FAZ	S	Cirrus Angioplex	Built in
Mameli et al.^29^	2019	1	94	53 (children)	–	41	VD	S, D	RTVue XR Avanti	Built in
Inanc et al.^30^	2019	1	117	60 (children)	–	57	VD, PD, FAZ	S, D	RTVue XR Avanti	Built in
Niestrata-Ortiz et al*.*^28^	2019	1	142	112 (children)	–	30	FAZ	S, D	DRI OCT-A Triton	Built in
Golebiewska et al.^27^	2017	1	248	188 (children)	–	60	VD, FAZ	S, D	RTVue XR Avanti	Built in
Dimitrova et al*.*^34^	2017	Both	62	*?*	29	33	VD, FAZ	S, D, CC	RTVue XR Avanti	Built in
Lupidi et al.^11^	2017	Both	95	*?*	48	47	VD, PD, FAZ	S, D	Spectralis OCT2	Built in
Gozlan et al.^23^	2017	Both	58	9	49	-	PD, FAZ,	S, D	RTVue XR Avanti	Built in
De Carlo et al.^35^	2015	Both	82	*?*	61	21	MA, PD, FAZ	Undisclosed	RTVue XR Avanti	Built in
Vujosevic et al.^16^	2018	Both	117	*?*	83	34	VD, PD	S	DRI OCT-A Triton Plus	Custom - Image J
Vujosevic et al.^21^	2019	Both	111	38	55	18	VD, PD, FAZ	S, D	DRI OCT-A Triton	Custom - Image J
Rodrigues et al.^22^	2019	Both	101	18	83	-	VD, FAZ	S, D, CC	Angiovue System	Built in
Choi et al.^12^	2017	Both	152	*?*	89	63	VD, FAZ	S, M, D, CC	UHS-SSOCT (MIT, NEEC)	Research prototype
Rosen et al*.*^17^	2019	Both	153	9	104	40	PD, FAZ	S, D	RTVue XR Avanti	Built in
Nesper et al.^37^	2017	Both	181	*?*	137	44	VD, PD, FAZ	S, D, CC	RTVue XR Avanti	Built in
Tang et al.^13^	2017	Both	434	*?*	434	–	VD, FD, FAZ	S	DRI OCT-A Triton	Built in
Tang et al*.*^41^	2019	Both	447	*?*	447	–	VD, FAZ	S	DRI OCT-A Triton	Custom - MatLab
Ishibazawa et al.^4^	2015	Undisclosed	47	*?*	47	–	MA, PD	S, D	RTVue XR Avanti	Built in
Shen et al.^18^	2018	Undisclosed	90	*?*	49	41	VD	S	RTVue XR Avanti	Built in
Samara et al.^42^	2017	Undisclosed	118	*?*	84	34	VD, FAZ	S, D	RTVue XR Avanti	Built in
Mastropasqua et al.^19^	2019	Undisclosed	119	*?*	94	25	VD, PD	S, D, CC	PLEX Elite 9000	Built in
Conti et al.^14^	2018	Undisclosed	136	*?*	99	37	PD, FAZ	S, D, CC	RTVue XR Avanti	Built in
Onishi et al.^15^	2018	Undisclosed	180	*?*	136	44	VD, PD	S, M, D	RTVue XR Avanti	Built in
Yang et al.^20^	2019	Undisclosed	282	*?*	239	43	VD	CC	RTVue XR Avanti	Built in
Cao et al*.*^7^	2018	2	138	–	71	67	VD, PD, FAZ	S, D, CC	RTVue XR Avanti	Built in
Ting et al.^8^	2017	2	100	–	100	0	VD, FD	S, D	DRI OCT-A Triton	Built in
Tan et al.^43^	2019	2	212	–	126	86	VD	S, D	RTVue XR Avanti	Built in
Zeng et al*.*^9^	2019	2	170	–	137	33	VD	S, D	RTVue XR Avanti	Built in
Kim et al*.*^10^	2018	2	155	–	155	0	VD, PD, FAZ	S, D	Cirrus Angioplex	Custom - Image J
***This study***	***2020***	***1***	***1186***	***956***	**–**	***230***	***VD, PD, FAZ***	***S***	***CirrusAngioplex***	***Built in***

OCTA, optical coherence tomography angiography; DM, diabetes mellitus; VD, vessel density; PD, perfusion density; FAZ, foveal avascular zone; S, superficial; M, medium; D, deep; CC, choriocapillaris; RS, research software.

Note: The OCTA parameters may not have been described in the original papers exactly as those described in the table, as the terms have been unified for comparison purposes. Devices: Cirrus Angioplex, Carl Zeiss Meditec, Dublin, CA; PLEX Elite 9000, Carl Zeiss Meditec, Inc., Dublin, CA; RTVue XR Avanti, Optovue, Inc., Fremont, CA; Angiovue System, Optovue Inc., Fremont, CA; DRI OCT-A Triton Plus, Topcon Europe, Milano; Spectralis OCT2, Heidelberg Engineering, Heidelberg. (*= see *“References”* section).

One of the key issues when interpreting quantitative analysis of OCTA images is which retinal plexus is being evaluated, a feature that is strongly related to the OCT device used for the image capture and the segmentation algorithm used by the software of the machine. In this report, we have used an OCT device that allows measurements only in the SCP with the commercial built-in software. The role of the different plexuses in the pathophysiology of DR is also a controversial topic, and there are discrepancies in the literature. Again, for the reasons mentioned above, most of the current evidence about which plexus and OCTA parameter reflects better the status of the macula of patients with diabetes needs to be taken from type 2 DM studies. Agemy et al. reported that VD measurements were different in the SCP and DCP of diabetic eyes, meanwhile Coscas et al. did not find any significant differences between these two plexuses in patients with diabetes.^31^^,^^32^ These controversial findings may be explained at least in part for the small sample size of these studies, and it is still unclear whether both plexuses are or are not affected simultaneously, limiting the conclusions of these previous reports. Consistently with our findings, several previous studies have found a lower VD in patients with DR compared with healthy controls, as well as an association between lower VD and greater DR stages in both SCP and DCP.^33^^–^^35^ What is clinically meaningful is that OCTA has demonstrated the ability to distinguish between healthy eyes and eyes with different stages of DR severity, using automated algorithms to measure different flow parameters in the macula.^24^^,^^31^^,^^32^^,^^36^^–^^38^ In order to provide helpful data for this differentiation, cut off points for each individual OCTA parameter are suggested in this paper, based on the ROC curves presented for controls and patients with type 1 DM without and with DR.

FAZ alterations are commonly seen in patients with diabetes.^36^^–^^38^ Previous studies in type 2 DM have shown that diabetic eyes are more likely to have an enlarged FAZ compared to healthy control eyes.^34^^,^^35^ Consistently with that, in our study, we found that in patients with type 1 DM the FAZ perimeter and area were progressively larger in greater DR stages but decreased in proliferative diabetic retinopathy (PDR) cases, both in 3 × 3 and 6 × 6 mm captures. We hypothesize that this finding in PDR cases may be explained by the reduced retinal thickness of these eyes and the fact that most of our PDR cases were previously treated with laser PRP in the moment of the scan, raising the interesting hypothesis of central reperfusion after PRP laser treatment. However, this is a controversial topic, as a significant variability in the FAZ area has been described in healthy individuals, and, therefore, it is difficult to determine a threshold between FAZ normality and pathological enlargement in retinal diseases.^39^ For this reason, the FAZ area may not be a sensitive marker to establish whether a study eye is normal or presents a retinal disease, but in some retinal conditions, as in DR, it may be associated with greater DR stages. As previously described, in this paper, we suggest potential cut offs for each of the FAZ parameters in ROC curves for controls and diabetic eyes without and with DR, in order to provide clinically relevant data to the ophthalmic community. Interestingly, Kim et al. showed a significant correlation between FAZ irregularity and visual acuity (VA).^10^ This parameter, FAZ circularity, may be helpful to objectively quantify the perifoveal capillary ring disruption and may potentially be a better tool to establish the degree of microvascular damage in the FAZ, which is ultimately related to VA.

The main strength of this study is the large number of type 1 DM eyes included, which to date is the largest OCTA cohort published in these patients. Second, the fact of obtaining the data with the device built-in commercial software and no manual corrections present two additional benefits: to obtain reliable and objective data of the analyzed parameters and to be reproducible and generalizable to other groups, without the need to use a research software, unpractical in daily practice, and unavailable to all medical teams. Third, the inclusion of a relatively large number of controls also add significant value to the OCTA analysis performed and constitutes another strong point of this study. As previously mentioned, a potential limitation of the study is the inability of the commercial software of the device used to quantify vascular flow parameters of the DCP, which, according to some authors, may be affected earlier in the development of DR. Future analysis of our dataset using an automated research software will shed some light on this specific issue. Nevertheless, our results have already detected flow alterations in the SCP indicating that this plexus has a determining role in the DR pathophysiology from early stages of the disease. Another possible limitation of the study may be the small number of patients with DM with severe NPDR, due to the efficacy of the public DR screening program in Spain. As the majority of the study cohort presented the milder forms of the disease, the results of this study are especially representative of early DR stages with a relatively spared macular anatomy. This may have had a positive effect in the reliability of the OCTA-derived metrics obtained, with a low rate of artifacts compared to other reported series in pathological eyes.^40^ However, the early stages of DR are the ones that may present subclinical signs that are worth detecting as soon as possible, in order to allow early interventions and to stop the progression of the disease.

In conclusion, OCTA is able to detect microvascular changes in type 1 DM diabetic eyes, which are correlated with the DR stage independently of age and duration of DM disease. Quantitative parameters as VD, PD, and FAZ area, circularity, or perimeter may be objectively quantified in diabetic eyes with this noninvasive technology. This automated analysis reveals details of the perifoveal vascular network status in an objective way, translating subjective qualitative findings into objective data, a significant step forward to allow ophthalmologists the integration of meaningful retinal vascularity data in daily clinical practice. These findings suggest a major role of OCTA in the next future, as these microvascular changes may be detected in their preclinical status, before the first clinical signs of DR appear in direct fundus examination. Future studies will focus on the possibility of defining OCTA-derived parameter ranges for DR severity stage classification, risk assessment, and, maybe, response to treatment and monitorization based on these objective grounds.

## Supplementary Material

Supplement 1

Supplement 2
