# Supplementary material for: Optical Coherence Tomography Angiography in Type 1 Diabetes Mellitus. Report 1: Diabetic Retinopathy
Source: Transl Vis Sci Technol. 2020 Sep 30;9(10):34. doi: 10.1167/tvst.9.10.34 (PMC7533741; doi:10.1167/tvst.9.10.34)
Supplement: Supplement 1 [file tvst-9-10-34_s001.pdf]

Supplemental Table 1. Demographic and baseline characteristics of study patients according to diabetic retinopathy stage

Variable	Statistic	Total (n=507)	Control (n=102)	No DR (n=269)	Mild NPDR (n=109)	Moderate NPDR (n=17)	Severe NPDR (n=3)	Proliferative DR (n=7)	P-value for trend[a]
General characteristics									
Age (years)	Mean (SD)	40 (12.4)	43 (14.1)	37.8 (12.2)	41.1 (10.6)	43.5 (10.1)	49.1 (13.8)	53.3 (7.2)	0.128
	Median (IQR)	38.7 (30.2; 49.6)	41.1 (30.6; 55.3)	36.4 (27.3; 46.7)	40.5 (33.6; 48.3)	43.6 (37.2; 48.7)	50.9 (34.6; 62)	53.1 (46; 61)	
Sex (female)	n (%)	273 (53.8%)	62 (60.8%)	143 (53.2%)	56 (51.4%)	8 (47.1%)	1 (33.3%)	3 (42.9%)	0.091
Smoking habit	n (%)	89 (17.8%)	8 (8.3%)	53 (19.8%)	25 (22.9%)	3 (17.6%)	0 (0%)	0 (0%)	<0.001
Hypertension	n (%)	50 (10%)	8 (8.4%)	23 (8.6%)	12 (11%)	4 (23.5%)	2 (66.7%)	1 (14.3%)	<0.001
BMI (kg/m^2)	Mean (SD)	24.6 (3.8)	23.8 (3.6)	24.4 (3.6)	25.2 (4)	25.8 (4.6)	27.9 (4.7)	27.5 (4)	<0.001
	Median (IQR)	24 (21.9; 27)	23.3 (21.3; 25.7)	23.9 (21.9; 26.7)	24.5 (22.5; 27.3)	26.7 (22.5; 29.4)	25.8 (24.6; 33.3)	25.2 (24.2; 30.6)	
Diabetes-related clinical characterisitics									
DM duration (years)	Mean (SD)	20 (10.8)	0 (0)	16.4 (9.7)	26 (8.4)	30 (7.9)	36 (5.2)	36.9 (16.2)	<0.001
	Median (IQR)	19.5 (10.9; 27)	0 (0; 0)	16 (8.5; 22.1)	26.1 (20.7; 32.3)	28.5 (23.5; 34.9)	37.8 (30.1; 40)	43.2 (21; 48.7)	
Macrovascular complications									
- Cerebrovascular disease	n (%)	4 (0.8%)	0 (0%)	1 (0.4%)	1 (0.9%)	2 (11.8%)	0 (0%)	0 (0%)	0.015
- Ischemic heart disease	n (%)	4 (0.8%)	1 (1%)	1 (0.4%)	0 (0%)	1 (5.9%)	1 (33.3%)	0 (0%)	0.061
- Peripheral vascular disease	n (%)	3 (0.6%)	1 (1%)	1 (0.4%)	0 (0%)	0 (0%)	1 (33.3%)	0 (0%)	0.332
Treatment									
Insulin requirements (IU/kg/day)	Mean (SD)	0.5 (0.3)	0 (0)	0.6 (0.2)	0.6 (0.2)	0.8 (0.3)	0.5 (0.2)	0.6 (0.1)	<0.001
	Median (IQR)	0.5 (0.3; 0.7)	0 (0; 0)	0.6 (0.4; 0.8)	0.6 (0.5; 0.8)	0.7 (0.6; 0.9)	0.5 (0.4; 0.7)	0.6 (0.5; 0.7)	
Antihypertensive treatment	n (%)	48 (9.6%)	8 (8.3%)	20 (7.4%)	12 (11%)	4 (23.5%)	2 (66.7%)	2 (28.6%)	0.002
Hypolipemiant treatment	n (%)	5 (1%)	0 (0%)	3 (1.1%)	1 (0.9%)	1 (5.9%)	0 (0%)	0 (0%)	0.276
Antiplatelet treatment	n (%)	15 (3%)	1 (1%)	4 (1.5%)	4 (3.7%)	4 (23.5%)	1 (33.3%)	1 (14.3%)	<0.001
Laboratory tests									
HbA1c	Mean (SD)	7.2 (1.2)	5.4 (0.3)	7.4 (1)	7.7 (1)	7.5 (0.7)	8.4 (0.7)	8 (1.1)	<0.001
	Median (IQR)	7.2 (6.4; 7.8)	5.4 (5.2; 5.6)	7.3 (6.7; 7.8)	7.5 (7; 8.2)	7.4 (6.9; 8.2)	8.6 (7.6; 8.9)	7.9 (7.1; 8.8)	
Total cholesterol (mg/dL)	Mean (SD)	179.5 (31.4)	194.1 (32)	176.1 (30.6)	176.7 (31.3)	190.2 (29.7)	157 (28.6)	187.9 (17.8)	0.109
	Median (IQR)	178 (158; 199)	194 (174; 215)	177 (156; 193)	171 (155; 197)	188 (172; 214)	148 (134; 189)	192 (171; 206)	
LDL-c (mg/dL)	Mean (SD)	103.7 (26)	116 (30.8)	100.7 (24.9)	102.3 (23.4)	109.1 (23.1)	83.3 (23.1)	110.4 (22.7)	0.088
	Median (IQR)	101 (86; 120)	115 (94; 140)	99 (83; 117)	101 (88; 120)	112 (84; 131)	86 (59; 105)	98 (90; 132)	

Supplemental Table 1. Demographic and baseline characteristics of study patients according to diabetic retinopathy stage

Variable	Statistic	Total (n=507)	Control (n=102)	No DR (n=269)	Mild NPDR (n=109)	Moderate NPDR (n=17)	Severe NPDR (n=3)	Proliferative DR (n=7)	P-value for trend[a]
HDL-c (mg/dL)	Mean (SD)	58.9 (17)	56.5 (13.8)	59.9 (16.8)	57.8 (18.5)	63.6 (24.4)	53.7 (7.6)	56.3 (8.1)	0.811
	Median (IQR)	56 (47; 68)	56 (48; 67)	58 (48; 70)	54 (45; 68)	54 (47; 93)	57 (45; 59)	58 (50; 63)	
Triglycerides (md/dL)	Mean (SD)	87 (52.7)	112.8 (56.2)	81.1 (54.9)	82.9 (42.8)	87.1 (29.9)	100.7 (20.3)	105.6 (40.4)	0.114
	Median (IQR)	72 (55; 100)	104 (67; 141)	65 (51; 89)	72 (56; 98)	80 (63; 109)	91 (87; 124)	116 (76; 130)	
Hb (g/L)	Mean (SD)	141 (12.7)	136.6 (12.4)	141.5 (12.2)	142.8 (12.8)	142.8 (13.1)	138.7 (30)	136.6 (15.9)	0.096
	Median (IQR)	140 (132; 150)	135 (129; 144)	140 (133; 151)	143 (133.5; 152)	142 (131; 151)	145 (106; 165)	134 (123; 151)	
Hematocrite	Mean (SD)	0.9 (7.3)	0.4 (0)	1.2 (9.8)	0.4 (0)	0.4 (0)	0.4 (0.1)	0.4 (0)	0.768
	Median (IQR)	0.4 (0.4; 0.5)	0.4 (0.4; 0.4)	0.4 (0.4; 0.5)	0.4 (0.4; 0.5)	0.4 (0.4; 0.5)	0.4 (0.3; 0.5)	0.4 (0.4; 0.5)	
Platetets (10^9/L)	Mean (SD)	251.2 (57.4)	250.9 (52.8)	248.1 (58.4)	252.3 (56.7)	267.1 (56.2)	303.3 (60.3)	286 (68.4)	0.054
	Median (IQR)	248 (211; 289)	245 (207; 296)	247 (210; 286)	246.5 (208; 284.5)	262 (216; 306)	326 (235; 349)	277 (227; 325)	
Creatinine (mg/dL)	Mean (SD)	0.8 (0.2)	0.8 (0.2)	0.8 (0.2)	0.8 (0.2)	0.8 (0.2)	1.3 (0.7)	1 (0.2)	<0.001
	Median (IQR)	0.8 (0.7; 0.9)	0.7 (0.6; 0.9)	0.8 (0.7; 0.9)	0.8 (0.7; 0.9)	0.8 (0.6; 0.9)	1.1 (0.8; 2)	1 (0.8; 1.2)	
Albumin-to-creatinine ratio (mg/g)	Mean (SD)	9.8 (24.7)	9.6 (14.7)	8 (19.3)	9.7 (22)	12.9 (28.9)	104.3 (173.8)	28.1 (37.7)	0.001
	Median (IQR)	3 (2; 7)	4 (2; 10)	3 (2; 6)	3 (2; 9)	4.5 (2; 9)	7 (1; 305)	10 (8; 37)	

OCTA: optical coherence tomography angiography; DR: diabetic retinopathy; NPDR: non proliferative diabetic retinopathy; HbA1c: glycated haemoglobin (A1c); BMI: body mass index; DM: diabetes mellitus; IU: insulin units LDL-c: low density lipoprotein-cholesterol; HDL-c: high density lipoprotein-cholesterol; Hb: haemoglobin; SD: standard deviation; IQR: interquartile range.

[a] Linear regression for continuous variables or Mantel-Hansel test for categorical variables.
