# Supplementary material for: Optical Coherence Tomography Angiography in Type 1 Diabetes Mellitus. Report 1: Diabetic Retinopathy
Source: Transl Vis Sci Technol. 2020 Sep 30;9(10):34. doi: 10.1167/tvst.9.10.34 (PMC7533741; doi:10.1167/tvst.9.10.34)
Supplement: Supplement 2 [file tvst-9-10-34_s002.pdf]

Supplemental Table 2. Ocular characteristics of the study eyes according to diabetic retinopathy stage

Variable	Statistic		Total (n=1009)	Control (n=203)	No DR (n=539)	Mild NPDR (n=214)	Moderate NPDR (n=34)	Severe NPDR (n=5)	Proliferative DR (n=14)	P-value for trend[a]
Visual acuity	n		1005	200	539	214	34	4	14	<0.001
(LogMAR)	Mean (SD)		0.03 (0.05)	0.02 (0.04)	0.03 (0.05)	0.03 (0.05)	0.07 (0.1)	0.23 (0.25)	0.11 (0.08)	
	Median (IQR)		0.03 (0; 0.03)	0 (0; 0.03)	0.03 (0; 0.03)	0.03 (0; 0.05)	0.03 (0.03; 0.08)	0.13 (0.08; 0.38)	0.08 (0.03; 0.17)	
Intraocular Pressure	n		1006	201	539	214	34	4	14	<0.001
(mmHg)	Mean (SD)		15.9 (3.1)	14.8 (2.5)	15.9 (3.1)	16.8 (3.5)	17.6 (3.1)	18.5 (4.1)	14.9 (2.6)	
	Median (IQR)		16 (14; 18)	15 (13; 16)	16 (14; 18)	16 (15; 19)	18 (16; 20)	19 (15; 22)	14.5 (13; 17)	
Spherical equivalent	n		994	194	534	214	34	4	14	0.805
	Mean (SD)		-2.8 (67.4)	-0.4 (2.1)	-4.8 (91.9)	-0.7 (2.2)	-0.4 (2)	0.6 (2)	2.2 (1.1)	
	Median (IQR)		-0.4 (-1.6; 0.4)	-0.1 (-1.4; 0.8)	-0.5 (-1.8; 0.3)	-0.5 (-1.6; 0.4)	-0.6 (-1.6; 0.6)	0.2 (-1.1; 2.2)	2.4 (1.1; 2.8)	
Axial Length (mm)	n		998	198	535	213	34	4	14	0.018
	Mean (SD)		23.7 (2.8)	23.8 (1.1)	23.8 (3.7)	23.4 (1.2)	22.9 (0.9)	22.7 (0.4)	22.9 (0.8)	
	Median (IQR)		23.5 (22.8; 24.3)	23.7 (23; 24.6)	23.5 (22.9; 24.4)	23.3 (22.8; 24)	23.2 (22.4; 23.6)	22.8 (22.5; 23)	22.7 (22.1; 23.6)	
Ocular pathology*	n (%)	No	1006 (100%)	201 (100%)	539 (100%)	214 (100%)	34 (100%)	4 (100%)	14 (100%)	-
Focal/Grid laser*	n (%)	No	1006 (100%)	201 (100%)	539 (100%)	214 (100%)	34 (100%)	4 (100%)	14 (100%)	-
PRP laser	n (%)	No	993 (98.7%)	201 (100%)	539 (100%)	214 (100%)	32 (94.1%)	4 (100%)	3 (21.4%)	<0.001
		Yes	13 (1.3%)	0 (0%)	0 (0%)	0 (0%)	2 (5.9%)	0 (0%)	11 (78.6%)	
Ocular surgeries*	n (%)	No	1006 (100%)	201 (100%)	539 (100%)	214 (100%)	34 (100%)	4 (100%)	14 (100%)	-
Ocular treatment*	n (%)	No	1006 (100%)	201 (100%)	539 (100%)	214 (100%)	34 (100%)	4 (100%)	14 (100%)	-

DR: diabetic retinopathy; NPDR: non-proliferative diabetic retinopathy; PRP: panretinalphotocoagulation SD: standard deviation; IQR: interquartile range.

[a] Linear regression for continuous variables or Mantel-Hansel test for categorical variables. *No cases with previous ocular pathology, previous macular laser (focal/grid), previous ocular surgery, or previously treated with intravitreal therapy of any kind were included in this study.
